# Evaluation and Dietary Exposure Assessment of Selected Toxic Trace Elements in Durum Wheat (*Triticum durum*) Imported into the Italian Market: Six Years of Official Controls

**DOI:** 10.3390/foods10040775

**Published:** 2021-04-04

**Authors:** Ciro Pompa, Teresa D’Amore, Oto Miedico, Chiara Preite, Antonio Eugenio Chiaravalle

**Affiliations:** Department of Chemistry, Istituto Zooprofilattico Sperimentale della Puglia e della Basilicata, Via Manfredonia 20, 71121 Foggia, Italy; ciro.pompa@izspb.it (C.P.); oto.miedico@izspb.it (O.M.); preite.chiara.pc@gmail.com (C.P.); eugenio.chiaravalle@izspb.it (A.E.C.)

**Keywords:** toxic trace elements, lead, cadmium, mercury, durum wheat, inductively coupled plasma-mass spectrometry, monitoring, dietary exposure

## Abstract

Durum wheat grains, which are mostly used for the production of pasta and several baked goods, represent a main source of vegetable proteins and calories. Concurrently, many contaminants, including toxic trace elements, may accumulate in them, posing a potential severe hazard to human health. In this context, for official control and food safety purposes, 346 samples of whole durum wheat imported into the Italian market from six countries (Australia, Canada, Kazakhstan, Russia, Turkey, and the United States) during the period 2015–2020 were analysed for cadmium (Cd), lead (Pb), and mercury (Hg) content using inductively coupled plasma mass spectrometry (ICP-MS). All the analysed samples were compliant with Food Agriculture Organization–World Health Organization and European Union regulations. The mean values were 0.0322 and 0.0162 mg kg^−1^, respectively, for Cd and Pb, while all samples showed levels below the limit of detection (0.004 mg kg^−1^) for Hg. The results were construed in terms of seasonality, year, and country of production, and compared with reference tolerance values. Confirming previous exposure studies, the obtained data and the dietary intake assessment showed that durum wheat-based products may have a significant impact on exposure to Pb and Cd (20–50%) in the overall population, particularly in more sensitive and/or exposed subgroups (infants, toddlers, and females).

## 1. Introduction

According to the Organization for Economic Co-operation and Development (OECD), the Food and Agriculture Organization (FAO) and the World Health Organization (WHO), wheat represents the most important source of food calories and vegetable macronutrients (including proteins, with a mean content of 15%, carbohydrates, and fat) and micronutrients (vitamins and oligoelements) at the global level. This cereal is the basis of all regional diets in the world. Moreover, it is the most commonly traded cereal and the food crop that covers the largest share of the global cultivated area [1,2,3]. In particular, durum wheat (*Triticum durum* or *Triticum turgidum* subsp. *durum* Desf; fam *Poaceae*) is the second most cultivated species of wheat, and constitutes the main ingredient of pasta, steamed/parboiled/roasted groats (e.g., bulgur, couscous, freekeh), and several types of baked goods (bread, pizza, biscuits etc.) [4]. The European Union (EU) is the leading producer of wheat, generating an average of 150.4 million tonnes per annum in the last 5 years. Among the EU member states, France, Spain, and Italy are the top manufacturers in terms of harvest and area of production. At the same time, Italy, which is the lead country in semolina production (with 76% of the mills in Europe) and pasta industry, is the main importer of durum wheat [3,5,6].

As the result of their widespread average consumption, within the vegetable and cereal category, durum wheat grains are under close surveillance, becoming a food safety issue as they represent a primary route of exposure to a wide variety of contaminants (mycotoxins, vegetal toxins, toxic trace elements, pesticides, and phthalic acid esters), that may influence not only nutritional quality and safety but also yield growth. This evidence justifies the considerable attention given to wheat and wheat-based products in human health, toxicology, nutrition, environment, and even in the economic field [7,8].

The most serious concern related to durum wheat-based product consumption remains the dietary exposure to toxic trace elements (TTEs). These are very persistent and very bioaccumulative (vPvB) pollutants that may contaminate wheat crops through atmospheric deposition, irrigation with polluted water, and exposure to animal manures, agrochemicals, inorganic fertilisers, and other anthropogenic activities [9,10,11]. Minimally, contamination with these toxicants may also occur during the processing chain [12]. Lead (Pb), cadmium (Cd), and mercury (Hg) are the most abundant TTEs, and are potentially found in vegetables in ionic/inorganic forms. In particular, it has been observed that they have a bioconcentration factor (BCF, an indicator of the tendency to accumulate into an organism) higher in durum wheat grains than in other cereals [13,14].

Even at low concentrations, TTEs may constitute a relevant hazard to human health. Like most TTEs, Pb, Cd, and Hg have a high affinity for the sulfhydryl groups of proteins, with a consequent ability to interfere with the function of hundreds of enzymes in the biological system [15]. Cd is classified as carcinogenic to humans (Group 1) by the International Agency for Research on Cancer (IARC), and as carcinogenic, reprotoxic, and suspected mutagenic (CRM) by the European Chemicals Agency (ECHA) [16,17]. Cd acts as biochemical function interfering-agent, mimetic of essential elements such as zinc and calcium (with a similar ionic radius and electronegativity), inductor of oxidative stress, and inhibitor of DNA repair. It thereby leads to chronic kidney disease (CKD) and lung, prostate, and kidney cancer [18,19]. Pb is considered as probably carcinogenic to humans (Group 2A, IARC) and toxic to reproduction (R, ECHA) [20,21]. Similarly, it competes and interferes with Ca^2+^ and Zn^2+^ ions, binding receptors, channels, transporters, and enzymes. This, together with its organotropism in the brain, bones, and adipose tissue, is responsible for lead-correlated disorders, which are particularly severe for infants and young children [22,23]. Finally, the degree of toxicity of Hg is related to its chemical species, which include elemental mercury (**^0^**Hg), inorganic mercury (iHg), and organic mercury (MeHg), with the latter being the most toxic species and mainly present in seafood products [24,25]. In vegetables and grains, where Hg assimilation is almost absent, the predominant chemical species is iHg. It can severely damage highly perfused tissues, for example in the gastrointestinal system, kidneys, and liver, thanks to its hydrophilicity [26,27].

Due to these remarkable toxic effects, these contaminants are strictly monitored, and new data and reports are steadily being acquired by food inspection and health regulatory agencies. In the FAO/WHO Codex Alimentarius (General Standard for Contaminants and Toxins in Food and Feed) and in European Regulation No. 1881/2006 there are established maximum levels (MLs) for Pb and Cd of 0.200 mg kg^−1^ in wheat. The MLs are reduced to 0.050 for Pb and 0.040 mg kg^−1^ for Cd in processed cereal-based foods and baby foods for infants and young children [28,29]. In contrast, there are no direct MLs for Hg in vegetables and cereals. However, European Regulation No. 73/2018 establishes Maximum Residue Levels (MRLs) for Hg compounds expressed as total Hg in several products, including wheat (MRL = 0.010 mg kg^−1^) [30]. 

In this context, this monitoring and evaluation study covers six years (2015–2020) of official controls on imported whole durum wheat (346 samples). Pb, Cd, and Hg contents were analysed by inductively coupled plasma mass spectrometry (ICP-MS) according to the official method EN 15763:2009 [31]. The results were examined through an inductive approach and discussed, with a particular emphasis on temporal, seasonal, and regional differences, and an evaluation of several exposure scenarios (age- and gender- related, and dietary habits-correlated) is provided.

## 2. Materials and Methods

### 2.1. Sample Collection and Preparation

The 346 samples of whole durum wheat were imported into the Italian market from 6 countries (Australia, Canada, Kazakhstan, Russia, Turkey, and the United States) and were intended for the manufacture of products for human nutrition. The analyses were carried out by the Istituto Zooprofilattico Sperimentale della Puglia e della Basilicata (IZS-PB) over six years (2015–2020) for official control purposes. The territorially competent authorities (the Port, Airport, and Border Health Offices and Border Inspection Posts) collected samples in agreement with European regulation No. 333/2007 and the standard method ISO 24333:2009 “Cereals and cereal products—Sampling”. Each wheat sample intended for laboratory test weighed about 10 kg and was representative of a lot of 10–500 tonnes [32,33]. A minimum of 50 samples and a maximum of 92 per year were analysed. In 2020, due to the COVID-19 pandemic and the economic and governmental consequences (exportation reductions/bans, economic recession, lower production rates, etc.) only 14 samples arrived at the IZS-PB [34,35]. 

### 2.2. Chemicals and Working Standard Solutions

High-purity reagents, nitric acid (trace element grade HNO_3_, 68% *v/v*), hydrogen peroxide (H_2_O_2_ 30% *v/v*), and ultrapure water (18.2 MΩ cm, at 25 °C) were supplied by Romil Ltd. (Cambridge, UK). Single-element standard solutions for Pb, Cd, and Hg at the concentration of 1000 mg L^−1^ were purchased from VWR International Ltd. (Leicestershire, UK). Working solutions were prepared by dilution in 2% (*v/v*) HNO_3_. Ultrapure argon (Ar, 99.9999% purity) was obtained from SAPIO s.r.l. (Monza, MI, Italy). The Standard Reference Material (SRM 1567b, wheat flour), supplied by the National Institute of Standards and Technology (NIST), was used for quality control and assurance.

### 2.3. Analytical Procedure and Quality Control

The sample preparation procedure, following a standard method (EN 13805:2014 “Determination of trace elements–Digestion under pressure”) consisted of microwave-assisted wet digestion in acid and oxidant conditions (6 mL of 68% (*v/v*) HNO_3_ and 2 mL of 30% (*v/v*) H_2_O_2_) with 1.00 g ± 0.0001 g of ground sample previously aliquoted into a Teflon vessel by using an analytical balance (Mettler Toledo s.p.a., Novate Milanese, Milan, Italy) [36]. An Ethos-One Microwave Reaction System (Milestone s.r.l. Sorisole, Bergamo, Italy) was used with the following program: (1) temperature increase up to 120 °C in 15 min, maintained constant for 10 min; (2) temperature increase up to 190 °C in 15 min, maintained constant for 20 min; and (3) cooling stage of about 30 min to reach room temperature.

The final solution (about 5% HNO_3_) was obtained by a 50-mL dilution with ultrapure water. The standard method EN 15763:2009 “Determination of arsenic, cadmium, mercury and lead in food” was used as the reference method for ICP-MS quantitative analysis [31]. From 2015 to 2017 the PerkinElmer Elan DRC II instrument was used. For analytical procedure details, please refer to Miedico et al. (2020) [37]. From 2018 to 2020 the analyses were performed by using the PerkinElmer NexION 2000 apparatus, which was equipped with a concentric nebulizer, a demountable quartz torch with a 2.0 mm internal diameter quartz injector tube, and a quadrupole ion deflector. The following operational parameters were set—nebulizer gas (Ar) flow rate: 1.01 L min^−1^; plasma gas (Ar) flow rate: 15 L min^−1^; auxiliary gas flow rate: 1.2 L min^−1^; radio frequency power: 1600 W. 

Rhodium and bismuth (both at 200 ng mL^−1^) were used as internal standards and added to standard and sample solutions by on-line mixing. One or more stable isotopes were monitored (^111^Cd, the sum of ^200^Hg and ^202^Hg, and the sum of ^206^Pb, ^207^Pb and ^208^Pb) in order to eliminate the intrinsic variability of isotope distribution and/or to improve sensitivity. A summary of validation parameters of the analytical procedure is reported in Table 1. The limits of detection (LODs) and limits of quantification (LOQs) were calculated as 3.3 and 10 times the standard deviation of 10 blank determinations, respectively, following the standard method EN 15763:2009. For quality control purposes (verification of precision and accuracy), the wheat flour SRM 1567b–NIST was used and treated similarly to the test samples throughout the analysis. For the recovery test of Pb and Hg the SRM was spiked to 0.020 mg kg^−1^ (Table 1).

Two replicates of each sample were analysed, and the trace element concentrations were evaluated as the mean of the two replicates.

### 2.4. Statistical Analysis

Elemental concentrations in durum wheat samples were checked for normality using the Kolmogorov–Smirnov test. Data were not normally distributed (*Critical D-value*: 0.073) for Cd, so in addition to mean and standard deviation values the median, minimum, and maximum values were also calculated. The normality test was not performed for Pb, since about 70% of samples were below the limit of quantification (<LOQ), nor for Hg since it was not detectable (<LOD) in all samples. Additionally, one-way analysis of variance (ANOVA) was used to compare the average contents between the samples grouped by season, year, and country of origin. Results were considered statistically significant at a *p*-value of <0.05. The MetaboAnalyst 5.0 web application, based on the R computing system (version “R v3.6.3” of February 2020, Xia Lab, Montreal, QC, Canada), was used for statistical evaluations [38].

For statistical analysis, the results of Pb and Cd were normalized by autoscaling (mean-centred and divided by the standard deviation of each variable). Moreover, for data statistical analysis, analytical results below the limit of quantification (<LOQ) were imputed as LOQ ½ for Pb and Cd. This substitution approach for treating left-censored data, commonly known as the “middle bound” (MB), may make the application of statistical techniques more difficult, but it permits avoiding the under- and/or over-estimation of contamination levels. In fact, it is more cautious than upper bound and lower bound approaches, i.e., using the value of LOQ and zero, respectively, to input results <LOQ [39].

## 3. Results and Discussion

### 3.1. General Considerations and Comparison with Previous Studies

Elemental concentrations in the 346 durum wheat samples are reported in Supplementary Table S1. Of the 346 samples, 103 (30%) had quantifiable values of Pb, while in 243 samples the values were <LOQ. Only two samples exceeded the maximum level of 0.200 mg kg^−1^; however, they were compliant, taking into account the uncertainty estimation in agreement with European Commission Regulation No. 333/2007 [32]. Similarly, Cd was quantifiable in 337 samples (97%) and <LOQ in nine samples. No sample was above the EU/FAO–WHO regulatory limits for Cd. Six (2%) and 17 (5%) samples for Pb and Cd, respectively, showed levels higher than 0.100 mg kg^−1^, which can be considered as a level of moderate concern (half of the wheat ML and twice the infant cereal-based product limit). 

Hg concentrations in all 346 samples were <LOD (0.004 mg kg^−1^). These results were compliant with European Commission Regulation No. 73/2018, since they were below the MRL of mercury-based compounds used for phytosanitary purposes [30]. Thus, in agreement with the most of reports and field studies, cereals and vegetables are not considered to be a primary exposure source of Hg [26,27,40]. Additionally, the ban of phytosanitary products containing mercury-based compounds in Europe and in other extra-EU countries is having a positive impact on the mitigation of environmental Hg pollution [41]. 

The current results have been compared with several surveys conducted all around the world concerning risk exposure assessment, monitoring, and total diet studies (TDS). The common thread of them is that the intake of Cd and Pb from wheat products may represent up to 40–50% of total dietary exposure of these toxicants [42,43,44,45]. However, it must be underlined that in most of these studies no distinctions were made between soft (*Triticum aestivum* Linn; fam *Poaceae*), durum, and other wheat grains, and therefore the authors decided to not include them in this study. An accurate distinction is more common in Italian studies. As an example, Conti et al. analysed four trace elements in 417 samples soft (178) and durum (239) wheat. Mean contents of 0.040 for Cd (range 0.013–0.094 mg kg^−1^) and 0.016 mg kg^−1^ for Pb (range 0.006–0.047 mg kg^−1^) were found in soft wheat grain. Similarly, the mean concentrations of Cd and Pb in durum wheat were 0.042 (range 0.011–0.088 mg kg^−1^) and 0.015 mg kg^−1^ (range 0.006–0.040 mg kg^−1^), respectively [42]. Analogously, the ICP-MS technique was used for the determination of Cd and Pb in selected food matrices including durum wheat semolina; the mean concentrations of Cd and Pb were 0.026 and 0.002 mg kg^−1^, respectively [43]. In Finland, the researchers found low levels of Pb in pasta samples produced with imported durum wheat (mean value: 0.018 mg kg^−1^; range 0.008–0.066 mg kg^−1^), while Cd concentrations were significantly higher, with a mean content of 0.079 mg kg^−1^ and a range of 0.026–0.182 mg kg^−1^ [44]. A Belgian study on Cd intake in an adult population showed that cereal products and potatoes were the main food groups contributing to Cd exposure. In 38 durum wheat pasta samples the mean concentration of Cd was 0.060 mg kg^−1^, with a range of 0.011–0.130 and a median of 0.054 mg kg^−1^ [45].

Table 2 and Table 3 summarize the overall mean concentrations of Cd (0.0322 mg kg^−1^) and Pb (0.0162 mg kg ^−1^) in durum wheat grains analysed over the last six years. Further considerations are needed, taking into account the variability associated with the year and country of production, as discussed in the next paragraphs. On the contrary, no significant differences emerged on the basis of seasonality, and subsequently only poor evaluations could be carried out. A possible delay between the harvesting and the exportation time, due to an undetermined stocking phase, and also a lack of accurate information about the period of harvest may mask any possible differentiation.

### 3.2. Cadmium Levels–Seasonal, Regional, and Temporal Variations

In Table 2 the results of Cd contamination in the overall 346 samples and in samples grouped by year, season, and country of production are presented.

**Figure 1:**
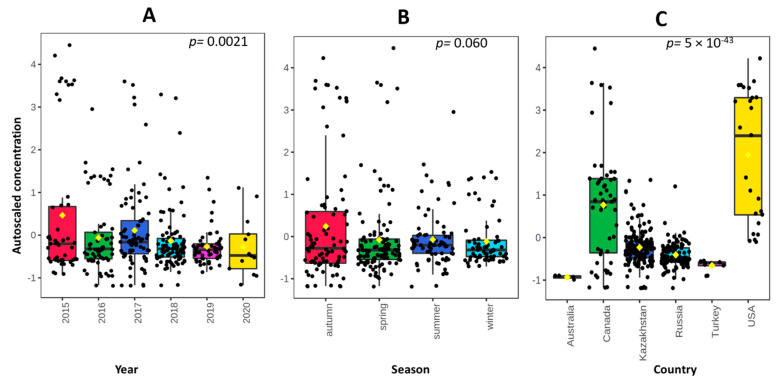
Boxplot representation of autoscaled values of Cd: (**A**) year of production (*p* = 0.0021); (**B**) season of production (*p* = 0.060); (**C**) country of production (*p* = 5 × 10^−43^).

Figure 1 shows a boxplot representation of normalized values of Cd in the same groups of the samples. On the basis of the ANOVA test, a significant variance (*p* = 0.0021) in normalized results was recorded for the year of production. A decreasing tendency can be observed from 2015 to 2020, with the exception of 2017. The samples collected and analysed in this year showed a mean Cd content of 0.0352 mg kg^−1^, which was significantly higher than the previous year and the following year (Figure 1A). Five samples (6.2% of the total in 2017) had a remarkably higher Cd concentration (from 0.099 to 0.125 mg kg^−1^); those five samples were all imported from the United States within a short period of time (November 2017). No other information was available from the sampling report of those five samples. The variance of Cd content in year groups is comparable, with a coefficient of variation (CV) of about 70%, apart from 2015 (CV = 95%) and 2019 (CV = 44%). A plausible reason is that in 2015 the samples came from five different countries, while in 2019, 92% of the samples came from two countries (Russia and Kazakhstan). A poor variance (ANOVA test, *p* = 0.060) in normalized results was observed for season of production/importation: the higher average in the autumn season (0.0385 mg kg^−1^) compared with the other three seasons is correlated with greater variability (standard deviation equal to 0.036). Moreover, as shown in plot B of Figure 1, all the samples with a Cd content higher than 0.100 mg kg^−1^ were clustered in the autumn and spring seasons.

In Table 2 and in the boxplot in Figure 1C, the grouping of the samples by country of production allows for the expression of some considerations by showing significant variance (*p* = 5 × 10^−43^). Four samples from Australia had very low levels of contamination that were only slightly above the LOQ. Similarly, the samples from Turkey had a low content of Cd. Russia and Kazakhstan were the two countries with the largest number of exported samples. This confirms the OECD/FAO data showing that Russian Federation is emerging as a major player in the international wheat market [1]. They had a similar Cd content and a comparable range. Furthermore, as shown in Figure 1C, the samples from Russia and Kazakhstan had a limited dispersion of 37% and 42%, respectively. On the contrary, the samples from Canada and especially those from the United States had much higher values. 

It is well documented that Cd accumulation in durum wheat grains may be affected by many factors, including growth conditions, geographical climate and microclimate, water availability, agricultural activities, durum wheat genotypes, and even handling and storage practices [46]. Generally, the data presented seem to be in agreement with the statement that durum wheat cultivated in warm and dry zones (e.g., Arizona, California, Saskatchewan, etc.), may have a higher Cd uptake than crops from predominantly cold–rainy and clay soil zones (e.g., Kazakhstan) [47,48].

### 3.3. Lead Levels–Seasonal, Regional, and Temporal Variations

Table 3 reports the Pb levels in all 346 samples, together with the range values and the samples grouped by year, season, and country of production. 

The statistical analysis for Pb is limited due to the high number of samples (70% of the total) with concentrations < LOQ. The MB substitution method, chosen by authors for treating these left-censored data, may have further reduced the variability of results compared with other censored data analysis methods (e.g., log-probit regression, maximum likelihood estimation, and non-parametric methods). However, it is widely used and highly recommended in the European Food Safety Authority (EFSA) guidelines “Management of left-censored data in dietary exposure assessment of chemical substances” [39]. 

**Figure 2:**
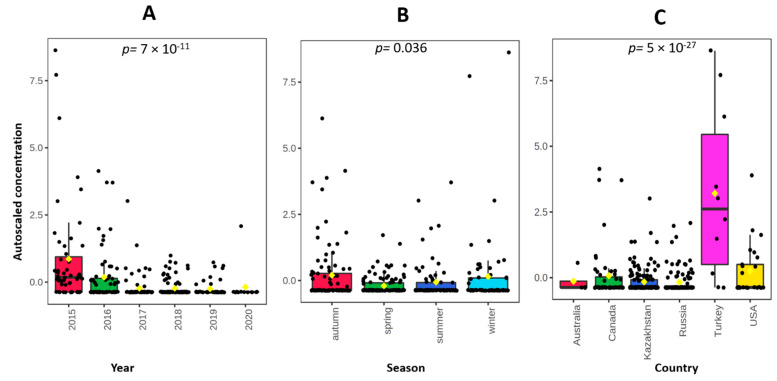
Boxplot representation of autoscaled values of Pb. (**A**) Year of production (*p* = 7 × 10^−11^); (**B**) Season of production (*p* = 0.036); (**C**) Country of production (*p* = 5 × 10^−27^).

Nevertheless, the observation of the data and of the boxplot graphs (Figure 2) leads to some findings. From 2015 to 2017 a decreasing trend is observable, while during the following three years a stationary linear trend was recorded (Figure 2A). Likely, the bioremediation policy is having a good impact on pollutant release into the environment [49].

Three samples with higher Pb levels were all imported from Turkey during the winter season of 2015. Despite the limited number of samples (*n* = 10), wheat grains from Turkey showed a mean level of Pb 10 times higher than that of other countries. In one sample imported from the United States the Pb concentration was higher than the level of moderate concern (0.123 mg kg^−1^), similarly to three samples from Canada (value about 0.120 mg kg^−1^). Correspondingly to the observations for Cd, grains from Russia and Kazakhstan, which were more representative, had mean Pb concentrations lower than those of other countries, with a low dispersion (CV = 112%). Finally, 75% of durum wheat from Australia had Pb concentrations < LOQ.

Much lower Pb values (about half) than Cd were expected, primarily keeping in mind the botanical characteristics of wheat plants, and excluding, with a good approximation assumption, the soil-borne contamination that arises in heavily polluted areas. In fact, Pb is massively retained by wheat roots and is not translocated in leaves and stems (low transfer coefficient), in contrast to Cd [7,50].

### 3.4. Dietary Exposure

From the mean concentrations of Cd and Pb found in the current monitoring study (0.0322 and 0.0162 mg kg^−1^, respectively) a possible exposure scenario was proposed for the Italian population. Dietary exposure to Pb and Cd was estimated using the food consumption data of pasta (fresh and dried) in Italy, which were obtained from the WHO/FAO collaborative platform for the different age categories [51,52,53]. These age groups included infants and toddlers (0–35 months), children (3–5 years), adolescents (6–14 years), young adults (15–49 years), older adults (50–74 years), and the elderly (≥75 years). In addition, dietary exposure was estimated for the entire female and male populations. As in most exposure assessment studies, the present discussion takes into account the dietary habits of “consumers only”, with the consumer considered as being a “subject who consumed at least one item within the food category on at least one eating occasion during the survey” [54]. It should be underlined that consumption data were not always available for dried and fresh pasta and for each population subgroup. Moreover, other wheat-derived food types such as bread and baked foods cannot be included in the present discussion due to the lack of information on the raw materials and ingredients. This assumption led to a marginal underestimation of Cd and Pb exposure levels, since a bulk part of harvested durum wheat is used for pasta production. Another important assumption necessary for the following considerations is that, along the durum wheat processing chain from grains to the final product, the concentrations of Cd and Pb do not vary substantially. Nevertheless, it is well known that Cd and Pb tend to accumulate in the external part of the kernel, the bran (the pericarp and aleurone layers). Thus, whole wheat products, despite their high content of fibre (wheat bran contains about 45% dietary fibre), may have greater levels of Pb and Cd [8,55,56,57,58].

The results are summarised in Table 4. The toxicological parameter of Estimated Weekly Intake (EWI), calculated by considering the mean value of Cd and Pb and the data on pasta consumption (the sum of dried and fresh pasta), was compared to the tolerable limits. The EFSA set the Tolerable Weekly Intake (TWI) for Cd at 2.5 µg kg^−1^ of body weight (b.w.) per week and the Provisional Tolerable Weekly Intake (PTWI) for Pb at 25 µg kg^−1^ of b.w. per week [24,48]. Two scenarios have been proposed for mean consumers and high consumers (95th percentile (P-95) consumers, whose diets consist of large amounts of pasta).

The Cd intake derived from pasta consumption for all consumers was 20% of the total exposure for average consumers and more than 34% for high consumers. The female population seemed to have greater exposure than males (approximately 20.8% of the TWI for females and 16.6% for males). In the macro-age group of individuals aged 0–14 years, infants and toddlers were more exposed, with durum wheat consumption covering more than 20% and up to 40% for high consumers of EWI, probably due to a less varied diet. Low variability, in terms of EWI, was observable in the adult population. In fact, for the 15–49 and 50–74 year age groups, the exposure to Cd from pasta was less than 20% for the average consumers. In contrast, a slightly more noticeable difference in Cd exposure was seen in in P-95: 0.829 and 0.652 µg kg^−1^ of b.w. for the 15–49 and 50–74 year age groups, respectively. For the elderly, the lack of data on fresh pasta probably greatly influenced the final calculation. Values of 5.5% for average consumers and of 12.4% for high consumers are unlikely. However, most dietary assessment studies (e.g., the EFSA Scientific report on “Cadmium dietary exposure in the European population”) indicate almost comparable Cd exposure derived from grains and grain-based products for adults (16–64 years), the elderly (65–74 years), and the very elderly (≥75 years) [59].

These results, indicating that durum wheat products largely contribute to total dietary Cd exposure, are in good accordance with some exposure assessment studies [42,43,44,45,59,60]. The United Kingdom TDS concluded that cereals (miscellaneous cereals and bread) account for about 40% of total dietary Cd exposure [60]. A wholly alike result was obtained from a Belgian study in an adult population [45]. The broad food groups contributing most greatly to Cd exposure for the European population were also found to be grains and grain products (26.9%) according to the EFSA [59].

The comparison of these data may be not very consistent. In fact, very different consumption patterns of these products among countries should be taken into consideration. However, the statistical analysis proposed by EUROSTAT (European Statistical Office) indicated that, among European members states, there were no extensive differences in the average annual consumption per capita of wheat (over 100 kg per capita from bakery products and pasta) [61]. Thus, the current discussion may be extended, providing some general and quite accurate indications: almost a half of all Cd intake, when exclusively considering the cereals category, is ascribable to pasta consumption.

Pb intake from durum wheat pasta in terms of average EWI is almost half that of Cd EWI (0.249 and 0.495 µg kg^−1^ of b.w. for Pb and Cd, respectively), and only 1% of the PTWI when considering mean consumers (Table 4). In the EFSA report, Pb dietary exposure in the European populations was about 4.76 (4.06–5.46) µg kg^−1^ b.w. per week using the MB approach, covering only 25% of PTWI [62]. Again, grains and grain-based products were estimated as major contributing food categories to Pb exposure (16.1%). This percentage corresponds to 0.766 µg kg^−1^ b.w. per week, which coincides with 3.1% of PTWI. Thus, by matching the results, it may be affirmed that durum wheat pasta covers 32.5% of Pb exposure derived from cereals. The EFSA affirmed that a 31% reduction was observed in comparison to the previous exposure assessment. In addition, Pb levels were estimated to had been reduced by about 23% between 2003 and 2010 [62].

## 4. Conclusions

The present study reports the results from six years of official controls on whole durum wheat samples imported from different countries destined for the Italian market. In total, 346 samples were analysed for Pb, Cd, and Hg content by ICP-MS. The results of the statistical analyses and evaluations of temporal, seasonal, and regional variations were discussed. In general, looking upon this study and others conducted at a global level, Pb, Cd, and Hg levels seem to have had a decreasing tendency in recent years, probably due to the policies of soil remediation recently adopted by many countries. Moreover, a significant difference emerged among the six countries of importation: US and Canadian samples had higher concentrations of Cd, while samples from Turkey showed the highest Pb content. Several scenarios of dietary exposure from the consumption of the main wheat-derived foods (pasta) have been discussed for different gender and age groups. It was found that cereals and in particular durum wheat products represent the main sources of Cd and Pb exposure. Some population groups (infants, toddlers, and females) may be more sensitive and/or exposed. 

Finally, according to the FAO and OECD, wheat consumption is expected to increase 13% by 2027. This percentage may be higher when considering new trends in dietary habits both in the overall population and in several subgroups. For these reasons, the continuous surveillance of these products appears mandatory. Furthermore, new exposure studies, especially those focused on more sensitive groups, are required to support risk assessment surveys.

## Figures and Tables

**Table 1:** Validation parameters and reference material analysis.

Element	LOQ (mg kg^−1^)	Determination Coefficient (R^2^)	Standard Reference Material NIST 1567b–Wheat Flour (mg kg^−1^)			
			Certified Val. ± U	Measured Val. ± U	Recovery (%) ^c^	Method Uncertainty (%) ^d^
Pb	0.012	0.9982	0.0104 ± 0.0024	≤LOQ (0.012)	105.0 ^b^	21%
Cd	0.0040	0.9999	0.0254 ± 0.0009	0.0249 ± 0.0045	96.3	18%
Hg	0.012	0.9986	0.0005 ^a^	≤LOQ (0.012)	103.6 ^b^	22%

^a^ Non-certified value, information only. ^b^ Evaluated by spiking tests. ^c^ Concentrations in test samples were not corrected for the recovery factor (not significantly different from 100%). ^d^ Obtained from the reproducibility values of the standardized method. LOQ: limit of quantification. Val.: Value.

**Table 2:** Concentrations (mg kg^−1^) of Cd in 346 durum wheat samples grouped by year, season, and country of production.

Cadmium		Number of Samples	Mean	Standard Deviation	Median	Range (Min–Max)
**Overall**	Overall	346	0.0322	0.026	0.025	0.002–0.147
**Year**	2015	51	0.0442	0.042	0.027	0.009–0.147
	2016	61	0.0307	0.022	0.024	0.002–0.108
	2017	79	0.0352	0.026	0.026	0.002–0.125
	2018	92	0.0290	0.020	0.024	0.002–0.117
	2019	50	0.0253	0.011	0.024	0.010–0.067
	2020	13	0.0252	0.018	0.020	0.002–0.061
**Season**	Autumn	91	0.0385	0.036	0.025	0.002–0.141
	Summer	75	0.0305	0.018	0.027	0.002–0.108
	Winter	66	0.0292	0.015	0.024	0.014–0.072
	Spring	114	0.0302	0.025	0.024	0.002–0.147
**Country**	Australia	4	0.00825	0.00096	0.0085	0.007–0.009
	Canada	51	0.0520	0.035	0.054	0.002–0.147
	Kazakhstan	151	0.0263	0.011	0.025	0.002–0.067
	Russia	105	0.0218	0.008	0.020	0.002–0.063
	Turkey	10	0.0153	0.003	0.017	0.009–0.018
	USA	25	0.0824	0.040	0.094	0.030–0.141

**Table 3:** Concentrations (mg kg^−1^) of Pb in 346 durum wheat samples grouped by year, season, and country of production.

Lead		Number of Samples	Mean	Standard Deviation	Median	Range (Min–Max)
**Overall**	Overall	346	0.0162	0.027	0.006	0.006–0.249
**Year**	2015	51	0.0397	0.054	0.021	0.006–0.249
	2016	61	0.0201	0.029	0.006	0.006–0.130
	2017	79	0.0098	0.013	0.006	0.006–0.099
	2018	92	0.0100	0.009	0.006	0.006–0.043
	2019	50	0.0093	0.008	0.006	0.006–0.036
	2020	13	0.0112	0.019	0.006	0.006–0.073
**Season**	Autumn	91	0.0217	0.032	0.006	0.006–0.184
	Summer	75	0.0145	0.021	0.006	0.006–0.118
	Winter	66	0.0202	0.042	0.006	0.006–0.249
	Spring	114	0.0106	0.010	0.006	0.006–0.063
**Country**	Australia	4	0.0125	0.013	0.006	0.006–0.032
	Canada	51	0.0181	0.029	0.006	0.006–0.130
	Kazakhstan	151	0.0118	0.013	0.006	0.006–0.099
	Russia	105	0.0116	0.013	0.006	0.006–0.073
	Turkey	10	0.1045	0.090	0.088	0.006–0.249
	USA	25	0.0229	0.028	0.006	0.006–0.123

**Table 4:** Dietary exposure to Cd and Pb: contribution of pasta (dried and fresh) of mean consumers and P-95 consumers for different age groups.

	Mean Consumption g/week (per kg Body Weight)			Cd		Pb		P-95 ^d^			Cd		Pb	
Population Groups	Dried Durum Pasta	Fresh Durum Pasta	Total	EWI ^a^	%TWI ^b^	EWI ^a^	%PTWI ^c^	Dried Durum Pasta	Fresh Durum Pasta	Total	EWI ^a^	%TWI ^b^	EWI ^a^	%PTWI ^c^
All	5.38	10.0	15.4	0.495	19.8	0.249	1.00	12.3	14.1	26.4	0.850	34.0	0.428	1.7
Female	5.59	10.6	16.2	0.520	20.8	0.262	1.05	12.3	14.2	26.5	0.853	34.1	0.429	1.7
Male	5.13	7.78	12.9	0.416	16.6	0.209	0.84	12.5	7.78	20.2	0.652	26.1	0.328	1.3
>75 years	4.24	-	4.24	0.136	5.5	0.069	0.27	9.63	-	9.63	0.310	12.4	0.156	0.6
50–74 years	4.21	9.72	13.9	0.449	17.9	0.226	0.90	8.78	11.5	20.3	0.652	26.1	0.328	1.3
15–49 years	5.21	10.2	15.4	0.496	19.8	0.250	1.00	11.4	14.4	25.8	0.829	33.2	0.417	1.7
6–14 years	8.16	-	8.16	0.263	10.5	0.132	0.53	17.7	-	17.7	0.570	22.8	0.287	1.1
3–5 years	12.3	-	12.3	0.398	15.9	0.200	0.80	28.2	-	28.2	0.908	36.3	0.457	1.8
0–35 months	15.9	-	15.9	0.513	20.5	0.258	1.03	31.5	-	31.5	1.01	40.6	0.511	2.0

^a^ EWI: estimated weekly intake (μg kg^−1^. body weight per week); EWI = (mean concentration × total mean consumption). ^b^ %TWI: percentage of tolerable weekly intake (μg kg^−1^ body weight per week); %TWI = (EWI/TWI) × 100. ^c^ %PTWI: percentage of provisional tolerable weekly intake (μg kg^−1^ body weight per week); %PTWI = (EWI/PTWI) × 100. ^d^ P−95: high consumers at the 95th percentile. -: not available data.

## Data Availability

The data presented in this study are available within the article and in Supplementary Material (Table S1).

## Supplementary Materials

The following are available online at https://www.mdpi.com/article/10.3390/foods10040775/s1. Table S1: Concentration of Pb, Cd, and Hg in 346 samples of whole durum wheat imported into the Italian market from six countries during the period 2015–2020.
